# CDC42—A promising immune-related target in glioma

**DOI:** 10.3389/fnins.2023.1192766

**Published:** 2023-07-05

**Authors:** Tao Jiang, Xianwei Wang, Jiaming Huang, Dong Chen

**Affiliations:** ^1^Department of Neurosurgery, The Dalian Municipal Central Hospital, Dalian, China; ^2^Dalian Municipal Central Hospital, China Medical University, Shenyang, China

**Keywords:** bioinformatics analysis, survival analysis, immune infiltration, CDC42, glioma

## Abstract

Glioma is the worst prognostic neoplasm in the central nervous system. A polarity-regulating GTPase in cells, known as cell division cycle 42 (CdC42), has been proven to have its overactivation tightly connected to high tumor malignancy. The RNA-seq and protein expression of CDC42 in tumor and comparison tissues were analyzed based on the online tools; CDC42 was remarkably boosted in tumor tissues compared to normal controls. A total of 600 patients in the analysis set from The Cancer Genome Atlas (TCGA) database and 657 patients in the validation set from the Chinese Glioma Genome Atlas (CGGA) database were adopted. The expression of CDC42 in clinical features and biological functions of glioma was analyzed, including differential expression analysis, survival analysis, Gene Ontology (GO), Kyoto Encyclopedia of Genes and Genomes (KEGG) analysis, and immune infiltration analysis. The enrichment of CDC42 was shown to be strongly associated with poor prognosis and terrible clinical indexes of glioma, including higher World Health Organization scale grade, wild-type isocitrate dehydrogenase 1 expression, O6-methylguanine-DNA methyltransferase non-methylated status, and 1p19q non-codeletion status (*p* < 0.0001). Functional enrichment analysis showed that CDC42 was highly correlated with immune and inflammatory responses in glioma. Additionally, the concentration extent of CDC42 was closely related to immune infiltration, immune checkpoints, and regulatory T (Treg) cell markers (CD4, CD25, and CD127). All evidence suggested that CDC42 may be a potential target for glioma immunotherapy.

## 1. Introduction

The most common primary intracranial tumor is gliomas (Lapointe et al., 2018). Despite the widespread use of surgery, chemotherapy, and radiotherapy (Ostrom et al., 2018), glioma has a high probability of recurrence due to the residue of tumor and cancer stem cells, and the average integral survival time of glioma patients is < 15 months (Abdul et al., 2018). In recent years, tumor-promoting microenvironment regulation and targeted immunotherapy have provided novel approaches for the treatment of glioma (Wang et al., 2017). The development of immune targets, including programmed cell death 1 (PD1), cytotoxic T lymphocyte-associated protein 4 (CTLA4), and killer cell lectin-like receptor subfamily B member 1 (KLRB1) (Litak et al., 2019; Mathewson et al., 2021), lays the groundwork for further tumor-free treatment of glioma. Regulatory T (Treg) immune cells are essential for preventing autoimmune diseases, and their infiltration in the tumor microstructure is conducive to microenvironment alleviation, tumor swelling, invasion and metastasis, angiogenesis, and antitumor immunity suppression. Additionally, cancer tissues with a large number of Treg cells infiltrated participates in the adverse prognosis of patients (Tanaka and Sakaguchi, 2017). Cell division cycle 42 (CDC42) is a small G protein with GTPase activity belonging to the Rho protein family that regulates multiple signaling pathways and participates in multiple biological processes. These processes involve the formation of filamentous pseudopodia and the induction of finger-like protrusions, which play an indispensable role in cell migration, invasion, and metabolism (Lawson and Ridley, 2018). The targeted inhibition of CDC42 expression has been shown to release the antineoplastic ability of effector T cells (Kalim et al., 2022); therefore, more research into the mechanism and effectiveness of CDC42 in tumor evolution and extension is necessary for glioma-targeted therapy. The Cancer Genome Atlas (TCGA) and Genotype-Tissue Expression (GTEx) databases were adopted for the differential analysis of CDC42 expression in pan-cancer, and further glioma RNA-seq data and clinical information were downloaded from TCGA and Chinese Glioma Genome Atlas (CGGA) databases to explore the relationship between CDC42 and glioma development through a collection of bioinformatics and survival analysis. Then, the differential genes were screened to execute Gene Ontology (GO) and Kyoto Encyclopedia of Genes and Genomes (KEGG) enrichment analysis. The correlation between CDC42 expression and immune cells, immune-related pathways, or functions was revealed by single sample gene set enrichment analysis (ssGSEA). Finally, the link between CDC42 expression and several immune checkpoints (ICs) or regulatory T (Treg) cell markers were established by correlation analysis.

## 2. Materials and methods

### 2.1. Data sources

The RNA-sequencing (RNA-seq) and matching clinical data of 600 patients (patients with incomplete data were deleted) with glioma in TCGA were obtained from the Genomic Data Commons (https://gdc.cancer.gov/, accessed on 3 September 2022), and 657 patients were used as a validation set that gained from the public CGGA database (http://www.cgga.org.cn/, accessed on 3 September 2022). The Tumor IMmune Estimation Resource (TIMER, http://TIMER.cistrome.org/, accessed on 8 September 2022) database was used to analyze the interrelation of CDC42 expression between different tumors and controls. The GTEx (https://www.gtexportal.org) database was used to supplement normal controls of the corresponding tissues. The Clinical Proteome Tumor Analysis Consortium (CPTAC, https://proteomics.cancer.gov) database was adopted to supplement and discover the CDC42 total protein expression level in tumors and controls. The c5.go.v2022.1.Hs.symbols and c2.cp.kegg.v2022.1.Hs.symbols.gmt datasets were obtained from the Molecular Signatures Database (MSigDB, https://www.gsea-msigdb.org/, accessed on 8 September 2022).

### 2.2. Gene and protein expression analysis of pan-cancer

The TIMER 2.0 database is a comprehensive resource (Li et al., 2020). TCGA serves as a background database that includes gene expression data for 33 tumors, and CDC42 was submitted in the “Gene_DE” column with an affiliated exploration module of the TIMER 2.0 website. Then, CDC42 expression differences between tumors and adjacent tumor-free tissues for 33 cancer types from the TCGA database were analyzed. Considering the lack of normal tissue controls in the TCGA database, we supplemented the expression of CDC42 in TCGA tumors using the web-based tool Gene Expression Profiling Interactive Analysis (GEPIA, http://geppia.cancer-pku.cn/, accessed on 8 September 2022) (Tang et al., 2019) with matched TCGA normal group and GTEx data as controls. The following parameters were set as follows: log_2_FC cutoff of 0.5 and a *p*-value of 0.05. UALCAN (http://ualcan.path.uab.edu, accessed on 8 September 2022) is an interactive portal (Chandrashekar et al., 2017) to analyze CDC42 protein expression in tumor and normal samples using data from the Clinical Proteome Tumor Analysis Consortium (CPTAC, http://ualcan.path.uab.edu/Analysis~prot.html).

### 2.3. Analysis of CDC42 expression in gliomas

The profiles of relevant clinicopathological and molecular biological features of CDC42 expression in gliomas were analyzed from TCGA and CGGA databases, including age, sex, pathology, isocitrate dehydrogenase (IDH) mutation status, 1p/19q codeletion status, O6-methylguanine-DNA methyltransferase (MGMT) promoter methylation status, and the World Health Organization (WHO) grade. The relationship between various clinicopathological features and CDC42 expression was shown by heatmaps and box plots drawn by “pheatmap” (version 1.0.12) and “ggplot2” (version 3.4.2) R packages. The significance of the dissimilarity was examined by the unpaired *t*-test and a one-way ANOVA.

### 2.4. Survival analysis

The Kaplan–Meier (KM) curve was portrayed to estimate the prognostic value of CDC42 in glioma patients through TCGA and CGGA databases. With median survival as the cutoff value, patients were separated into two groups of low and high levels, and the results were visualized utilizing the “survminer” (version 0.4.9), “survival” (version 3.3-1), and “ggplot2” (version 3.4.2) R package. To evaluate the reliability of CDC42 expression in predicting survival, the receiver operating characteristic (ROC) curves were established using the “timeROC” (version 0.4) R package. Independent prognostically relevant factors were evaluated through univariate Cox and multivariate Cox proportional hazard regression models. The outcomes were displayed using the forest diagram produced by the R software.

### 2.5. Gene function enrichment analysis

The CDC42-combination proteins were obtained from the STRING online tool (https://string-db.org, accessed on 8 September 2022) (von Mering et al., 2003), and the functional annotations of each binding protein were downloaded. The CDC42 were entered in the “Proteins by name” query, and “Homo sapiens” were selected for organisms. Furthermore, the following main parameters were confirmed: network type (“full STRING network”), required score [“medium confidence (0.400)”], and size cutoff (“no more than 20 interactors”). The results were imported into the Cytoscape (Shannon et al., 2003) (version 3.7.2) software, and we applied the maximal clique centrality (MCC) algorithm to screen out the top four genes most closely related to CDC42. Based on the median CDC42 expression, samples were split into two groups in TCGA and CGGA databases, respectively. Differential expression genes (DEGs) were identified with the LIMMA (Ritchie et al., 2015) (version 3.52.4) R package after being analyzed (log_2_FC > 1.5, *p* < 0.01). GO and KEGG analyses were carried out using the “clusterProfiler” (version 4.4.4), “org.Hs.e.g.db” (version 3.15.0), “ggplot2” (version3.4.1), and “enrichplot” (version 1.16.2) R packages. In biological process (BP), cell component (CC), molecular function (MF), and KEGG, barplot and dot plot charts illustrated the most significant pathways in upregulated DEGs.

### 2.6. Immune infiltration and correlation analysis

Using the R packages “GSVA” (Hanzelmann et al., 2013) (version 1.44.5), “limma,” and “GSEABase” (version 1.58.0), ssGSEA was utilized to evaluate the RNA-seq data of 29 critical immune gene sets (Guo et al., 2022) from each glioma sample. Immune-related gene set scores between the two CDC42 expression groups were shown with the “pheatmap” package. The variances between both groups were demonstrated using the horizontal box plot manufactured by the “ggplot2” R package. We calculated the percentage of 22 types of tumor-infiltrating immune cells (TIICs) in the glioma microenvironment by applying the cell-type identification by estimating relative subsets of RNA transcripts (CIBERSORT) deconvolution algorithm (https://cibersortx.stanford.edu/) (Newman et al., 2015) to further explore the relationship between immune cell infiltration and CDC42 expression in gliomas. To illustrate the distribution of TIICs in the CDC42 high and low expression groups, boxplot and barplot were utilized by R software. The correlation between CDC42 and the relevant immune checkpoint was studied, and boxplot and scatter plots were employed by R software.

### 2.7. Statistical analysis

All statistical analyses were performed using R v4.2.1 and SPSS v.25.0 software (IBM Corp.). In the analysis of the correlation of clinicopathological features, the significance of the distinction between the two groups was tested by an unpaired *t*-test. One-way ANOVA was performed on multiple groups of samples to assess significant differences among three groups and above. The Kaplan–Meier survival analysis was performed to compare the overall survival of glioma patients with high and low CDC42 expression. The log-rank test was implemented to evaluate group differences. The Spearman correlation analysis was utilized to assess the correlation between groups of immune cells or immune-related pathways. All statistical tests were two-sided, and the cutoff for statistical significance was set at a *p*-value of <0.05.

## 3. Results

**Figure 1:**
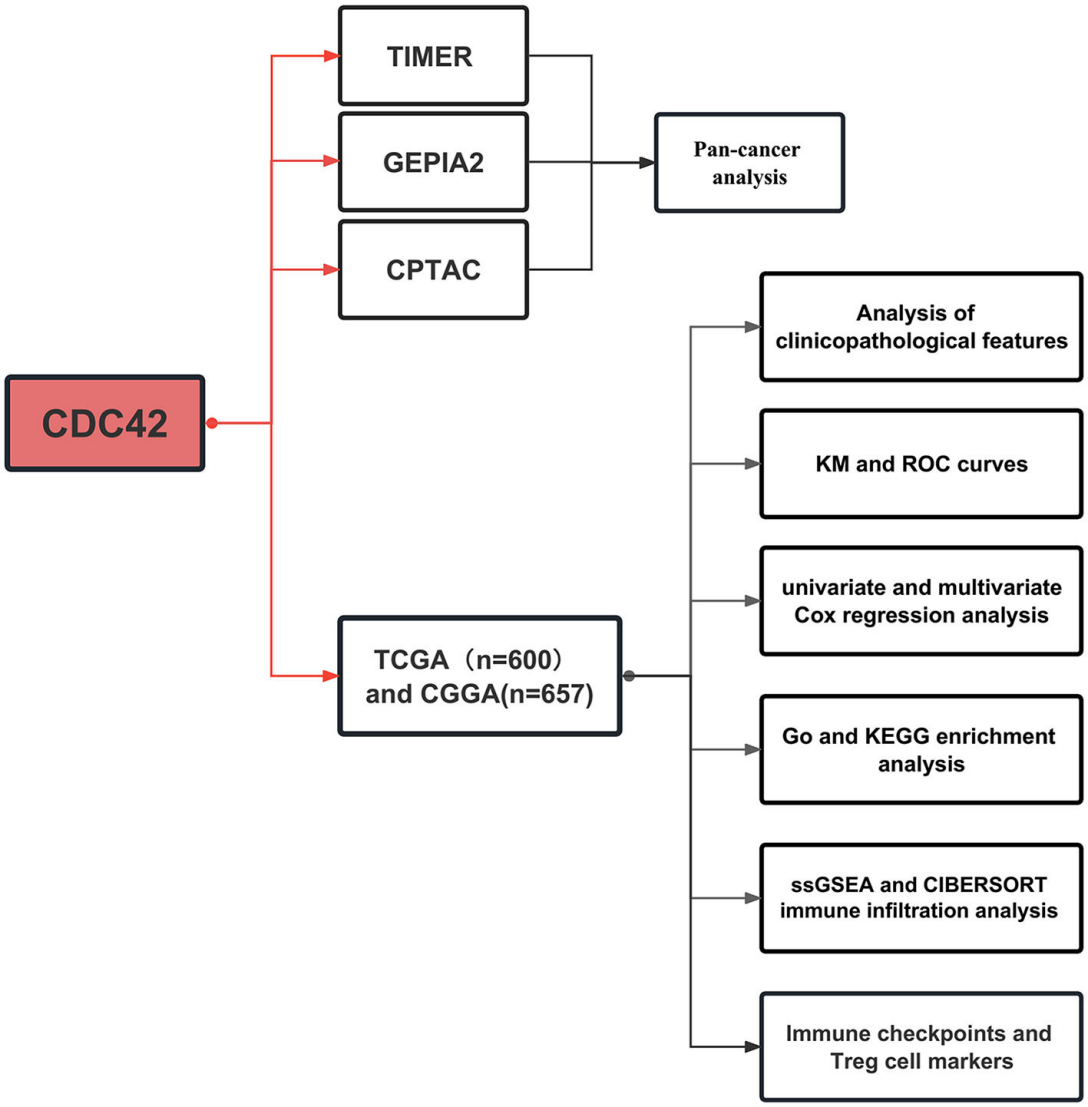
Process of this study. Pan-cancer analysis was carried out using three online tools based on The Cancer Genome Atlas (TCGA) and Genotype-Tissue Expression (GTEx) databases. TCGA databases and the Chinese Glioma Genome Atlas (CGGA) were employed for further glioma-related research.

Pan-cancer analysis was accomplished through the three online tools based on TCGA and GTEx databases, including mRNA and protein expression distinction between tumor and adjacent normal tissues in different organs. Subsequently, CDC42 expression levels in various clinical and molecular biological characteristics of glioma were explored through TCGA and CGGA databases. The functional enrichment of differential expression genes was analyzed, and the correlation between CDC42 and relevant immune cells, immune-related pathways, and immune checkpoints was investigated. The specific flow chart is shown in Figure 1.

### 3.1. CDC42 is enriched in human pan-cancer

**Figure 2:**
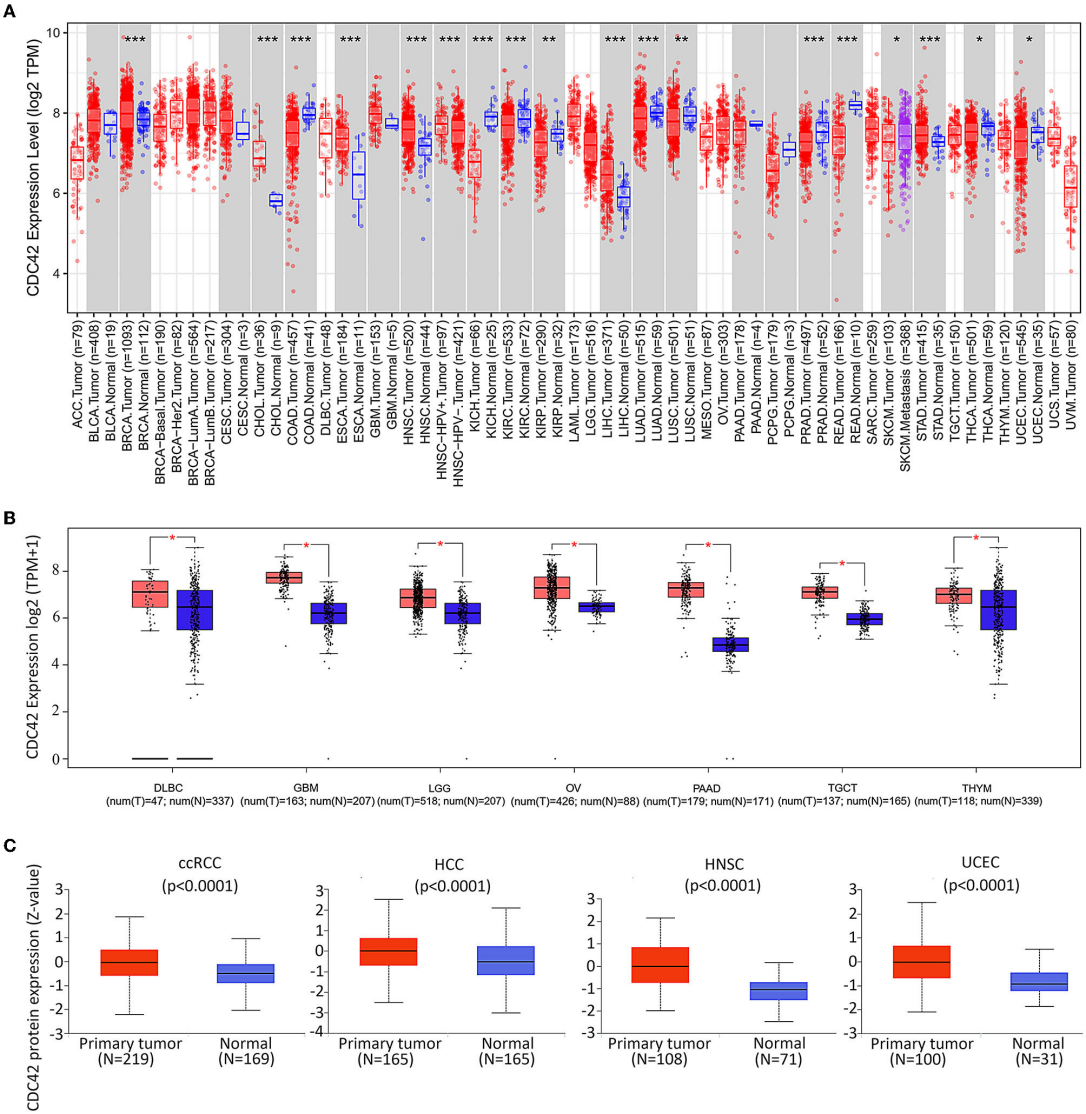
Expression levels of CDC42 in pan-cancer. **(A)** Human CDC42 expression levels in different cancer types from TCGA database in Tumor Immune Estimation Resource (TIMER). *p < 0.05, **p < 0.01, ***p < 0.001. *n*, number of samples; **(B)** For the type of DLBC, GBM, LGG, OA, PAAD, TGCT, and THYM in the TCGA project, the corresponding normal tissues of the TCGA and GTEx databases were included as controls. Data for the box plot were provided. T, tumor; *N*, normal. *p < 0.05; **(C)** CDC42 proteomic expression profile in ccRCC, HCC, HNSC, and UCEC from Clinical Proteome Tumor Analysis Consortium (CPTAC) samples. Standard deviations from the median for samples for a given cancer type are shown by z-values. *N*, number of samples.

TIMER2.0 was applied to study the mRNA expression levels of CDC42 for the entire 33 tumors in the TCGA database to explore the difference in CDC42 expression between tumors and adjacent tumor-free tissues. The outcome demonstrated that the expression rates are relatively high in breast invasive carcinoma (BRCA, *p* = 0.00073), cholangiocarcinoma (CHOL, *p* = 2.26e-09), esophageal carcinoma (ESCA, *p* = 6.60e-05), head and neck squamous cell carcinoma (HNSC, *p* = 1.63e-09), liver hepatocellular carcinoma (LIHC, *p* = 6.34e-11), and stomach adenocarcinoma (STAD, *p* = 0.00026) compared with the corresponding tumor-free tissues (Figure 2A). Interestingly, we did not find differences in expression between CDC42 normal tissue and tumors in some tumor types, such as GBM and LGG. We further supplemented the analysis using the GTEx database to consider the lack of normal sample controls in the TCGA database.

The GEPIA online tool was used to analyze tumors in the TCGA database and corresponding TCGA and GTEx normal tissues as controls. We found that CDC42 was highly expressed in large B-cell lymphoma (DLBC, *p* < 0.05), glioblastoma multiforme (GBM, *p* < 0.05), lower grade glioma (LGG, *p* < 0.05), ovarian cancer (OV, *p* < 0.05), pancreatic adenocarcinoma (PAAD, *p* < 0.05), testicular germ cell tumors (TGCTs, *p* < 0.05), and thymoma (THYM, *p* < 0.05) than normal tissues (Figure 2B).

The normal metabolism of tissues and various life activities is closely related to protein functions. The results of the CTPAC online tool implied that CDC42 protein was enriched in clear cell renal cell carcinoma (ccRCC, *p* = 1.7e-08), hepatocellular carcinoma (HCC, *p* = 1.0e-05), head and neck cancer (HNSC, 5.3e-18), and endometrioid cancer (UCEC, *p* = 1.5e-09) compared to normal tissues (Figure 2C). This further provided important evidence for the high expression of CDC42 in most tumor tissues. All evidence manifested that this molecule may be a promising marker for tumor-targeted therapy.

### 3.2. CDC42 is associated with glioma clinicopathological characteristics

**Figure 3:**
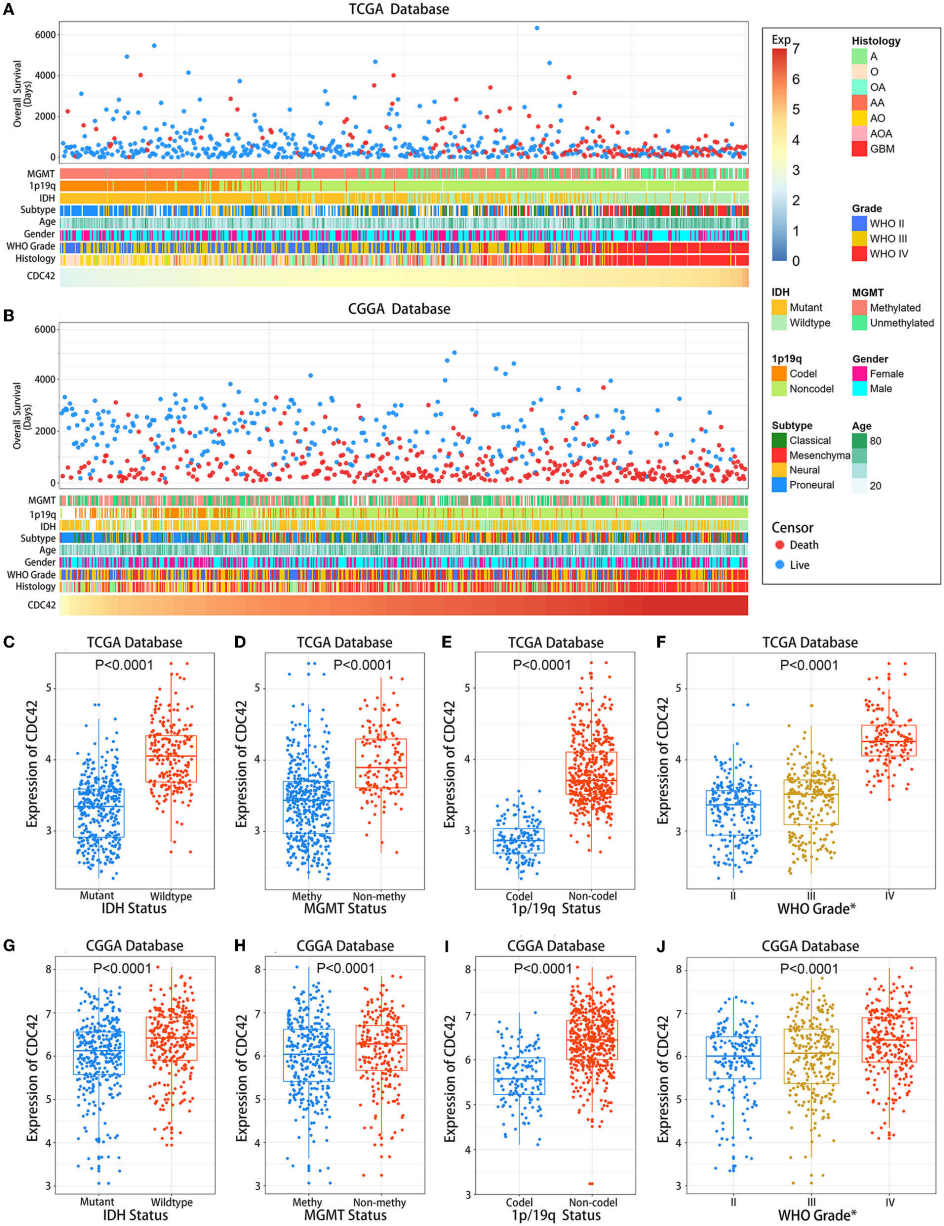
Association between CDC42 expression and clinicopathological characteristics of gliomas. **(A)** An overview of TCGA database glioma clinicopathological characteristics connected to CDC42; **(B)** An overview of CGGA database glioma clinicopathological characteristics connected to CDC42; **(C, G)** CDC42 has significantly increased in gliomas without isocitrate dehydrogenase (IDH) mutation in the TCGA and CGGA databases. An unpaired *t*-test was performed to determine the difference's significance; **(D, H)** CDC42 has increased in the O6-methylguanine-DNA methyltransferase (MGMT) promoter-unmethylated gliomas in the TCGA and CGGA databases. An unpaired *t*-test was performed to determine the difference's significance; **(E, I)** CDC42 was significantly increased in gliomas without 1p/19q codeletion in the TCGA and CGGA databases. An unpaired *t*-test was performed to determine the difference's significance; **(F, J)** CDC42 were significantly increased in higher-grade gliomas in TCGA and CGGA databases. One-way ANOVA was performed to determine the significant difference.

We then investigated the association between CDC42 expression levels and several clinicopathological features of glioma in a total of 1,257 glioma patients (Table 1) from the TCGA and CGGA databases, respectively. CDC42 showed distinct expression levels in glioma about their clinicopathological characteristics, such as IDH mutation status, MGMT promoter methylation status, 1p/19q codeletion status, pathological classification, and the WHO grade, which were analyzed in the TCGA database (Figure 3A) and validated in the CGGA database (Figure 3B). We found that CDC42 was highly enriched in high-grade glioma patients (Figures 3F, J), IDH wild-type glioma patients (Figures 3C, G), non-methylated glioma patients (Figures 3D, H), and non-codeletion glioma patients (Figures 3E, I) in TCGA and CGGA databases. The clinical prognoses of the patient and the degree of tumor malignancy are inextricably linked with the pathological classification of glioma (Ostrom et al., 2014). We discovered that dangerous pathological classifications of gliomas, such as glioblastoma, had high levels of CDC42 expression (Supplementary Figure 1).

**Table 1:** Characteristics of patients in TCGA and CGGA databases.

**Characteristic**	**TCGA**	**CGGA**
Samples	600	657
**Age (years)**		
≤ 40	232	268
>40	368	388
**Gender**		
Male	348	374
Female	252	283
**IDH status**		
Mutant	371	333
Wild type	223	276
**1p/19q codeletion status**		
Codeletion	149	137
Non-codeletion	446	454
**Grade** ※		
II	213	172
III	236	248
IV	151	237
**Histology** ※		
Astrocytoma	166	254
Oligodendroglioma	201	137
Oligoastrocytoma	82	29
Glioblastoma	151	237
**Fifth WHO classification**		
Astrocytoma, IDH-mutant, 1p/19q non-codeletion	221	192
Oligodendroglioma, IDH-mutant, 1p/19q codeletion	149	104
Glioblastoma, IDH-wildtype	138	182

※ According to the fourth WHO classification (WHO 2016 classification).

### 3.3. CDC42 highly expressed tumors have a significantly poor prognosis

**Figure 4:**
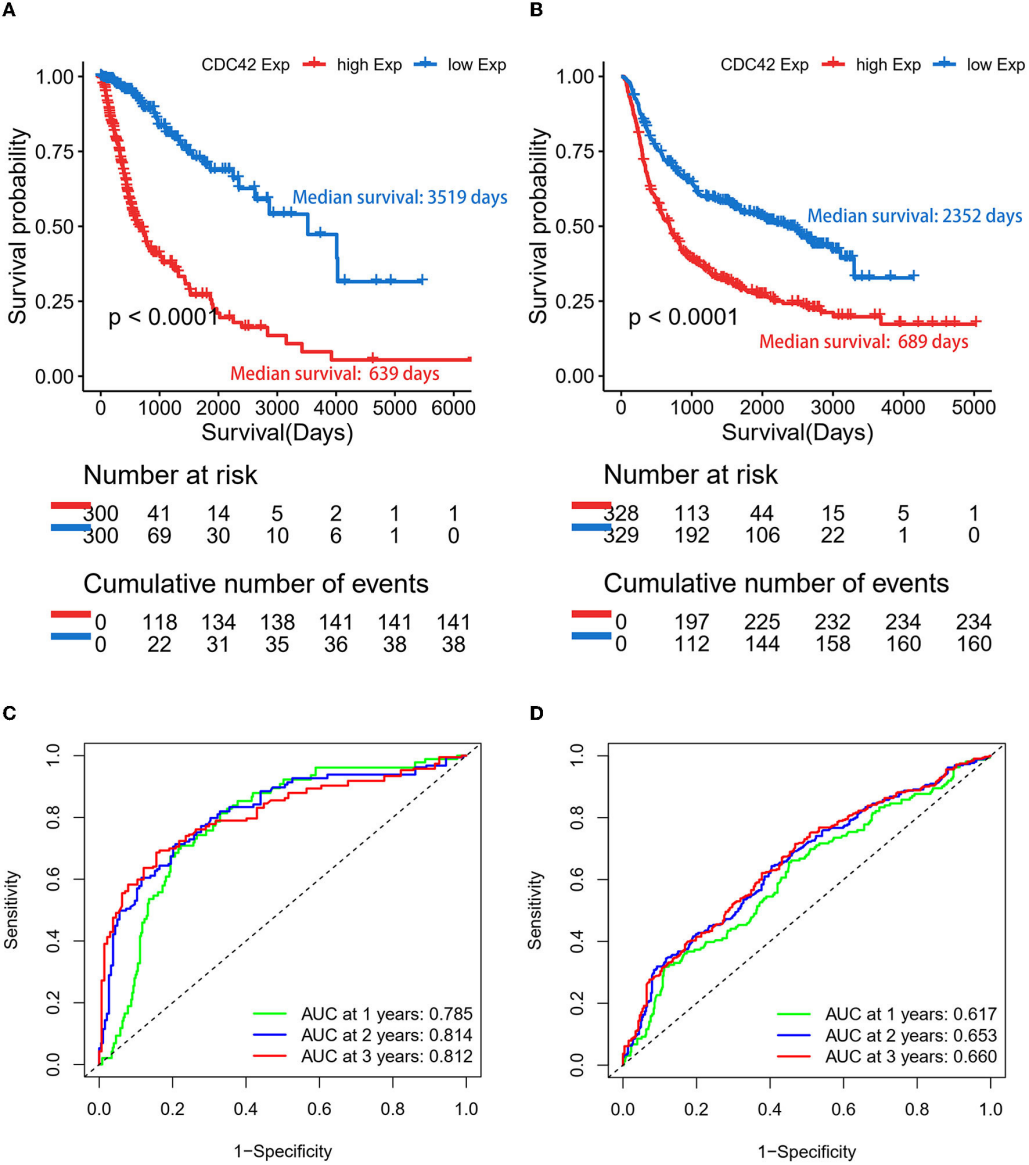
Gliomas with high expression of CDC42 have a poor prognosis. **(A, B)** CDC42 expression in gliomas according to the Kaplan–Meier curve in TCGA and CGGA datasets. The median expression of CDC42 serves as the group's cutoff point. A log-rank test was used to determine the significance of the prognostic value. **(C, D)** Time-dependent ROC curves for overall survival (OS) at 1, 2, and 3 years in TCGA and CGGA databases.

**Figure 5:**
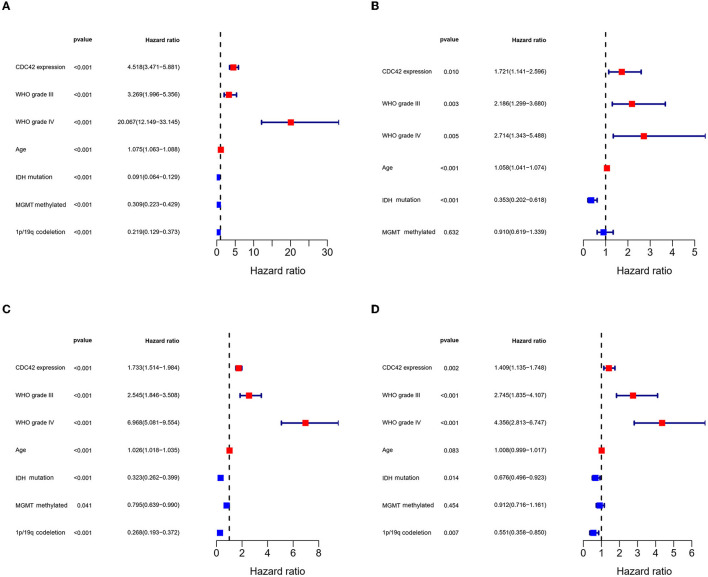
Cox regression analysis of factors related to glioma prognosis. **(A, C)** Univariate Cox analysis of factors affecting glioma overall survival in TCGA and CGGA databases. **(B, D)** Multivariate Cox analysis of factors affecting glioma overall survival in TCGA and CGGA databases.

According to an analysis of the KM curve based on the TCGA and CGGA databases, patients with high CDC42 expression (median survival 639 days) in the TCGA (Figure 4A) database had considerably shorter overall survival compared to patients with low CDC42 expression (median survival 3,519 days). This conclusion was verified in the CGGA database (Figure 4B). The ROC curves for 1, 2, and 3 years of survival were established, and the effectiveness of CDC42 in predicting glioma patient survival was confirmed by the area under curve (AUC) in TCGA (AUC at 1 year: 0.785, AUC at 2 years: 0.814, and AUC at 3 years: 0.812) (Figure 4C) and CGGA (AUC at 1 year: 0.617, AUC at 2 years: 0.653, and AUC at 3 years: 0.660) (Figure 4D) databases. CDC42 expression was a predictive factor in univariate and multivariate Cox regression studies, independent of other known prognostic markers, such as WHO grade, age at diagnosis, IDH mutation, 1p/19q codeletion, and MGMT promoter methylation (Tables 2, 3). In univariate Cox regression analysis, the forest plot demonstrated that high expression of CDC42 was linked to a shorter overall survival (HR = 4.518, 95% CI 3.417–5.881, *p* < 0.001) in the TCGA database, and a large number of other clinicopathologic variables, including WHO grade III (compared to grade II), grade IV (compared to grade III), age, IDH status, and 1p19q codeletion status, also showed significant relationships with overall survival (Figure 5A). Subsequently, multivariate Cox regression revealed that poor overall survival was associated with high expression of CDC42 (HR = 1.721, 95% CI 1.141–2.596, *p* < 0.01), WHO grade III (compared to grade II) (HR = 2.186, 95% CI 1.299–3.680, *p* < 0.01), WHO grade IV (compared to grade III) (HR = 2.714, 95% CI 1.343–5.488, *p* < 0.01), and older age (HR = 1.058, 95% CI 1.041–1.074, *p* < 0.01), while IDH mutation (HR = 0.353, 95% CI 0.202–0.618, *p* < 0.001) was connected with a beneficial outcome (Figure 5B). Similar results were displayed by the CGGA database (Figures 5C, D).

**Table 2:** Prognostic factors in the TCGA database: multivariate and univariate analyses of overall survival (OS).

**Variable**	**Univariate analysis**		**Multivariate analysis**	
	**HR (95% CI)**	***P*** **-value**	**HR (95% CI)**	***P*** **-value**
CDC42 expression	1.733 (1.514–1.984)	1.59E-15	1.409 (1.135–1.748)	0.002
WHO grade III	2.545 (1.846–3.508)	1.18E-08	2.745 (1.835–4.107)	9.01E-07
WHO grade IV	6.968 (5.081–9.554)	1.90E-33	4.356 (2.813–6.747)	4.28E-11
Age	1.026 (1.018–1.035)	1.37E-09	1.008 (0.999–1.017)	0.083
IDH mutation	0.323 (0.262–0.399)	3.38E-26	0.676 (0.496–0.923)	0.014
MGMT methylated	0.795 (0.639–0.990)	0.041	0.912 (0.716–1.161)	0.454
1p/19q codeletion	0.268 (0.193–0.372)	3.02E-15	0.551 (0.358–0.850)	0.007

CI, confidence interval; HR, hazard ratio; IDH, isocitrate dehydrogenase; WHO, World Health Organization; MGMT, O6-methylguanine-DNA methyltransferase.

**Table 3:** Prognostic factors in the TCGA database: multivariate and univariate analyses of overall survival (OS).

**Variable**	**Univariate analysis**		**Multivariate analysis**	
**HR (95% CI)**	***P*** **-value**	**HR (95% CI)**	***P*** **-value**
CDC42 expression	4.518 (3.417–5.881)	3.49E-29	1.721 (1.141–2.596)	9.65E-03
WHO grade III	3.269 (1.996–5.356)	2.56E-06	2.186 (1.299–3.680)	0.003
WHO grade IV	20.067 (12.149–33.145)	1.09E-31	2.714 (1.343–5.488)	0.005
Age	1.075 (1.063–1.088)	5.10E-34	1.058 (1.041–1.074)	1.39E-12
IDH mutation	0.091 (0.064–0.129)	2.56E-40	0.353 (0.202–0.618)	2.64E-04
MGMT methylated	0.309 (0.223–0.429)	1.94E-12	0.910 (0.619–1.339)	0.632
1p/19q codeletion	0.268 (0.193–0.372)	2.11E-08		

CI, confidence interval; HR, hazard ratio; IDH, isocitrate dehydrogenase; WHO, World Health Organization; MGMT, O6-methylguanine-DNA methyltransferase.

### 3.4. CDC42 is significantly associated with immune and inflammatory response

**Figure 6:**
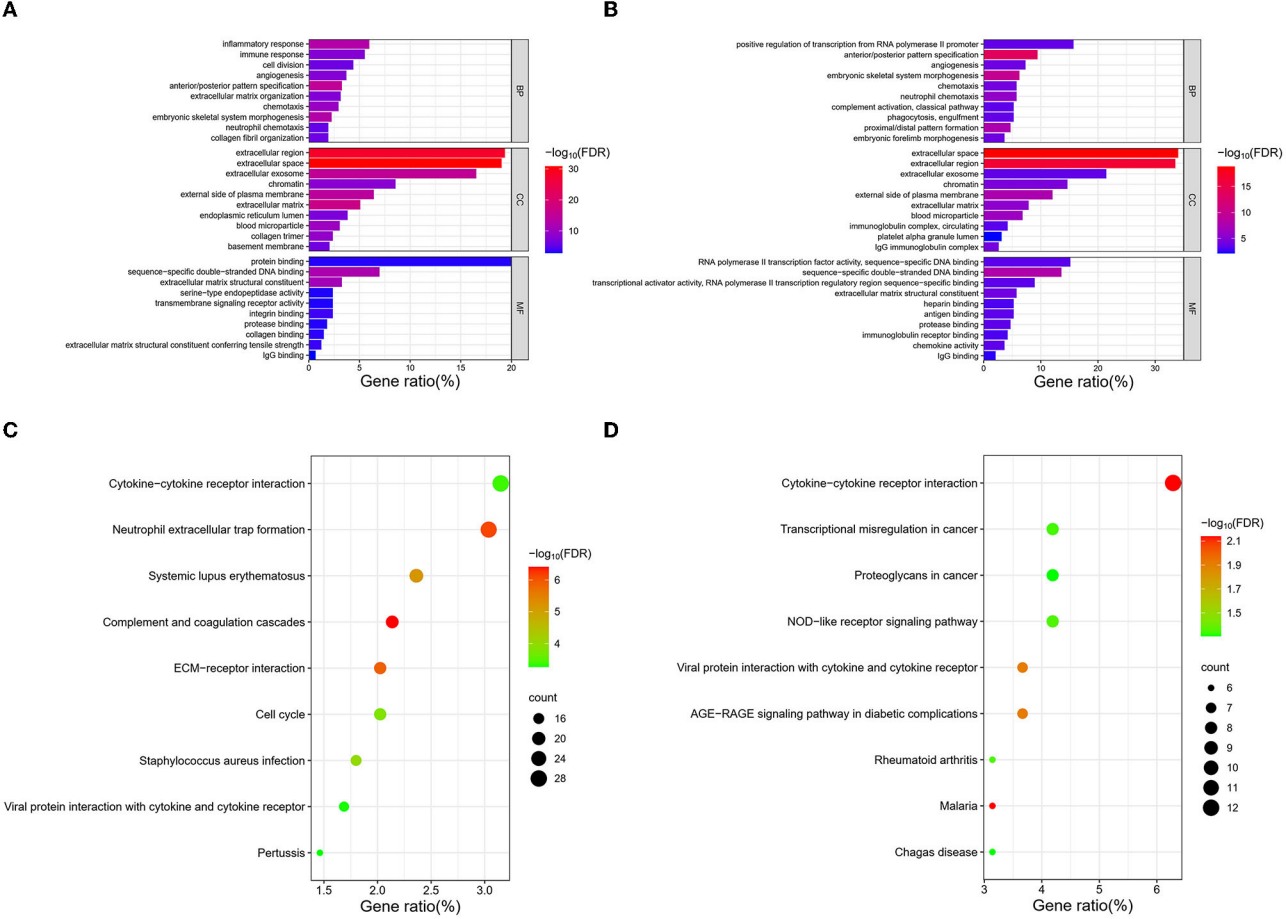
CDC42-related gene enrichment analysis. **(A, B)** GO analysis of DEGs associated with CDC42 in TCGA and CGGA databases. **(C, D)** KEGG enrichment analysis of DEGs associated with CDC42 in TCGA and CGGA databases.

A total of 20 CDC42-bound proteins were screened through the STRING online database (Supplementary Figure 2A). We imported the results into the Cytoscape software and identified the top four proteins (PAK1, ARHGDIA, IQGAP1, and WAS) that were most closely associated with CDC42 using applying the MCC algorithm (Supplementary Figure 2B). Overactivation of CDC42 can lead to an exaggerated of p21-activated kinases (PAKs), which is crucial in triggering the growth of tumors (Radu et al., 2014). Zhang et al. (2023) discovered that the tripartite motif containing 56 (TRIM56) acts through the IQGAP1-CDC42 signaling axis to promote glioma cell migration and invasion. This evidence suggests that CDC42 plays a role in the development of glioma. To further investigate the biological functions and molecular mechanism connection between CDC42 and tumors, differential analysis of genes between the high and low groups of CDC42 expression was performed using the “limma” package. We screened (log_2_FC = 1.5, *p* < 0.01) 1,011 and 226 DEGs associated with high expression of CDC42 in TCGA and CGGA databases for further GO and KEGG analyses. The top 20 upregulated and downregulated DEGs were shown with heatmaps (Supplementary Figures 3, 4). Barplots and dot plots were drawn to observe the enrichment results using the R software. GO analysis of TCGA and CGGA databases manifested that DEGs related to CDC42 high expression were intimately related to immune and inflammatory responses, chemotaxis, extracellular region, and chromatin (Figures 6A, B). KEGG enrichment analysis showed that CDC42 was associated with signaling pathways such as cytokine–cytokine receptor interaction, neutrophil extracellular trap formation, and transcriptional misregulation in cancer (Figures 6C, D). These results indicated that CDC42 may be crucial for immune and inflammatory responses and disease regulation in glioma.

### 3.5. CDC42 is highly correlated with immune infiltration in glioma

**Figure 7:**
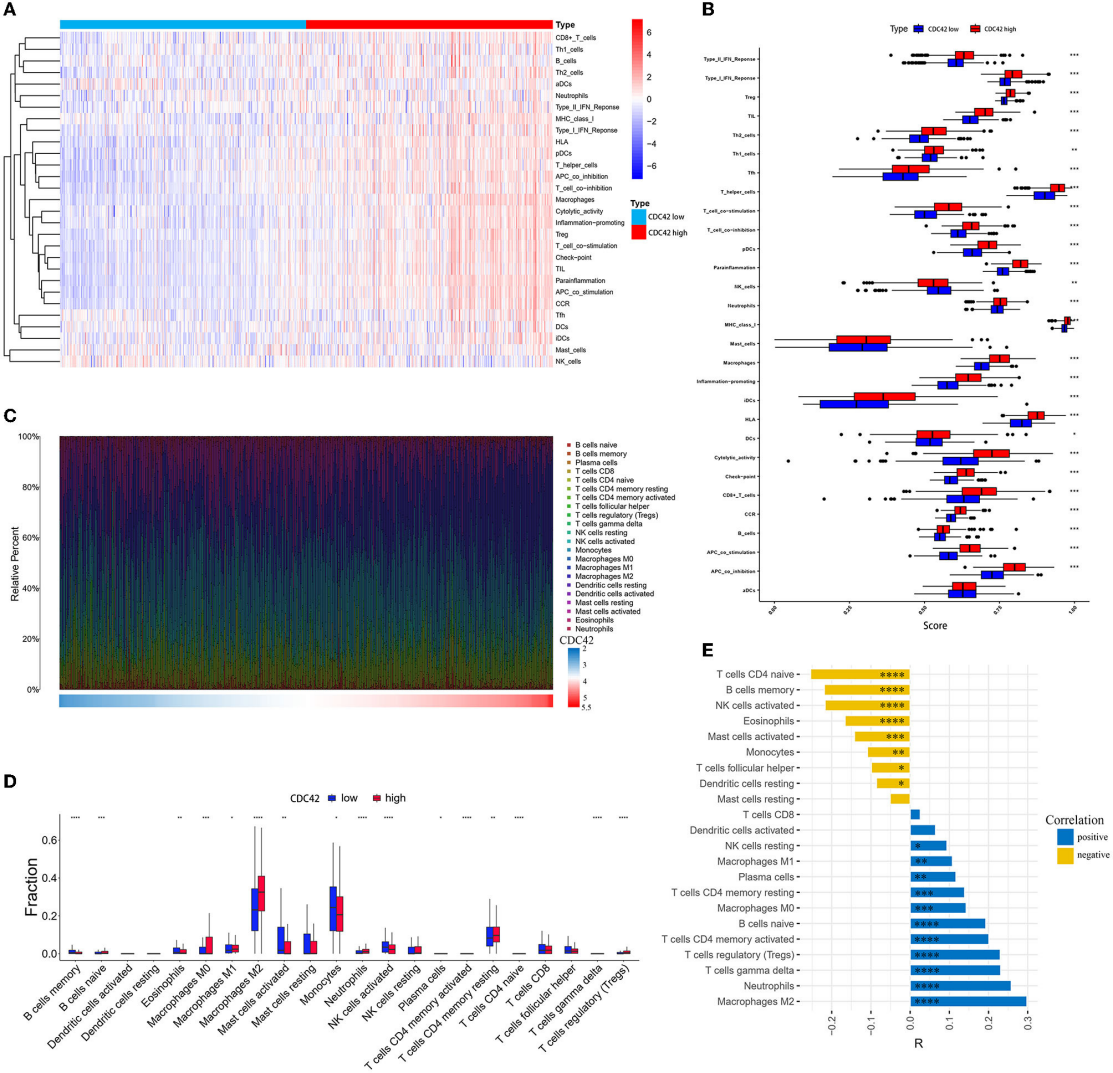
Immune cells, immune-related pathways, and functional analysis in the CDC42 high and low expression groups. **(A)** Heatmap of CDC42 expression and 29 well-established immune-associated gene sets based on ssGSEA in the TCGA database. **(B)** The horizontal box plot of 29 well-established immune-associated gene sets based on ssGSEA between the CDC42 high and low expression groups in the TCGA database. **(C)** The proportion of 22 immune cells based on CIBERSORT in the TCGA database. **(D)** The box plot of 22 immune cells based on CIBERSORT between the CDC42 high and low expression groups in the TCGA database. **(E)** Correlation analysis of CDC42 with 22 immune cells in the TCGA database. R, correlation coefficient. *p < 0.05, **p < 0.01, ***p < 0.001, ****p < 0.0001.

Based on the 29 fully established immune-associated gene sets (Supplementary Table 1), ssGSEA was applied to examine the association between CDC42 expression and immune cell types as well as immune-related pathways or functions in the TCGA database. The heat map indicated that CD8^+^ T cells, Treg cells, T helper (Th) cells, and macrophages were significantly infiltrated in CDC42 hyper-expression glioma samples, yet natural killer (NK) cells showed the opposite result (Figure 7A). In addition, immune-related pathways and functions such as type I and type II interferon (IFN) response, major histocompatibility complex (MHC) class I molecules, T cell co-inhibition and co-stimulation, marker genes for immune checkpoints, para-inflammatory factors, antigen-presenting cell (APC), co-inhibition and co-stimulation, and chemokine receptor (CCR) activation (Figure 7B) were involved with CDC42 high expression.

To further investigate the association between immune cells in glioma and CDC42 expression, we computed the infiltration ratio of 22 types of TIICs in the 2 groups of CDC42 high and low expression using the CIBERSORT algorithm. M0, M1, and M2 macrophages (*R* = 0.143, *p* < 0.001, *R* = 0.108, *p* < 0.01 and *R* = 0.289, *p* < 0.0001, respectively), neutrophils (*R* = 0.258, *p* < 0.0001), T cell CD4^+^ memory (resting and activated, *R* = 0.139 and *R* = 0.201, respectively, *p* < 0.001), Treg cells (*R* = 0.23, *p* < 0.0001), and B cells' naïve cells (*R* = 0.193, *p* < 0.0001) were highly infiltrated in CDC42 high expression group (Figures 7C, D). T cell CD4^+^ naive (*R* = −0.254, *p* < 0.0001), B cells memory (*R* = −0.219, *p* < 0.0001), NK cells active (*R* = −0.217, *p* < 0.0001), eosinophils (*R* = −0.166, *p* < 0.0001), and monocytes (*R* = −0.109, *p* < 0.01) exhibited the opposite result (Figure 7E).

### 3.6. High CDC42 expression is correlated with glioma immunotherapy

**Figure 8:**
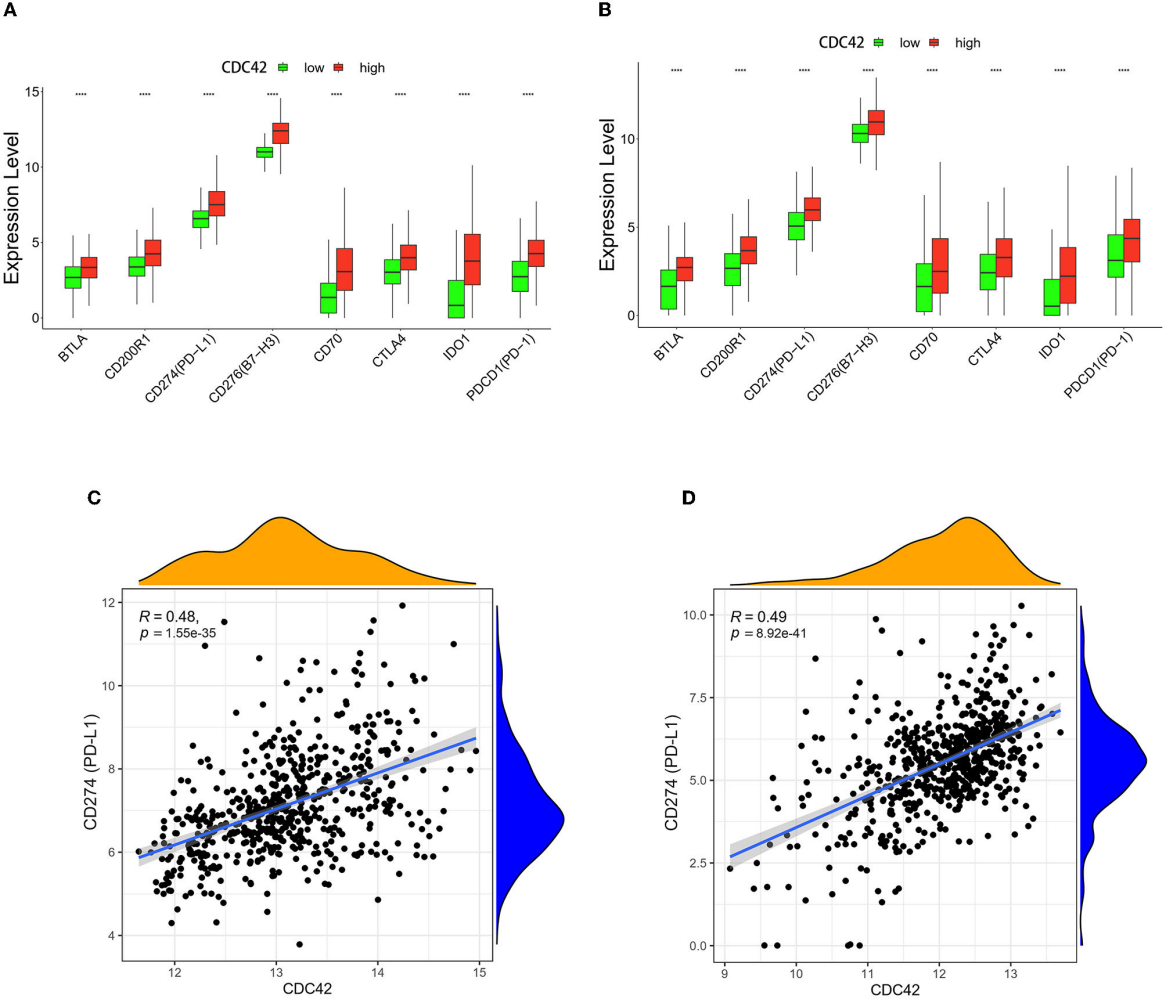
Correlation analysis between CDC42 expression and immune checkpoints. **(A, B)** Expression levels of PD-L1 and other immune checkpoints in high and low CDC42 expression groups in TCGA and CGGA databases. ****p < 0.0001. **(C, D)** Correlation analysis between CDC42 expression and PD-L1 in TCGA and CGGA databases.

**Figure 9:**
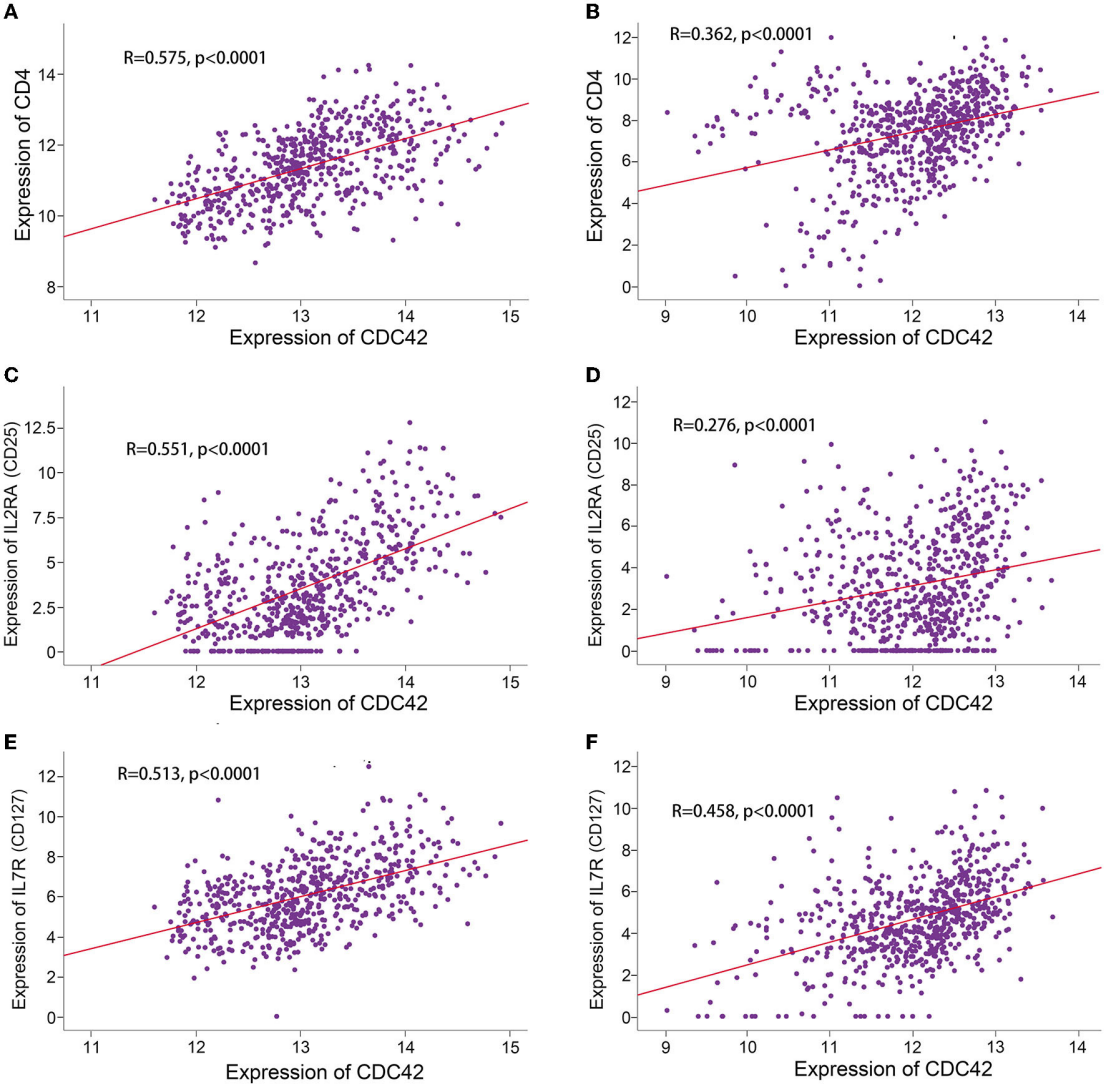
Correlation analysis between CDC42 expression and Treg cell markers. **(A, B)** Correlation analysis between CDC42 expression and CD4 in TCGA and CGGA databases. **(C, D)** Correlation analysis between CDC42 expression and IL2RA (CD25) in TCGA and CGGA databases. **(E, F)** Correlation analysis between CDC42 expression and IL7R (CD127) in TCGA and CGGA databases.

Immune cells express a class of immunosuppressive molecules known as immune checkpoints, which can control the level of immunological activation. Immune evasion of tumors can be accomplished by upregulating immune checkpoints (Di Giacomo et al., 2019). Immune checkpoint inhibitors (ICIs) have been applied in a range of cancer as a form of cancer immunotherapy based on this mechanism (Carlino et al., 2021). The effectiveness of anti-programmed cell death 1 (PD1) immunotherapy, a promising pathway (Wang et al., 2019) in the treatment of gliomas, is correlated with the expression of the programmed death ligand 1 (PD-L1) and the tumor immune microenvironment cell components (Yang et al., 2021; Yao et al., 2021). In TCGA and CGGA databases, we compared the expression levels of PD1, PD-L1, and 6 additional immune checkpoints in the CDC42 high and low expression groups. B and T lymphocyte attenuator (BTLA), CD200 receptor 1 (CD200R1), PD-L1, B7 homolog 3 (B7-H3), CD70, cytotoxic T lymphocyte-associated protein 4 (CTLA4), indoleamine-pyrrole 2,3-dioxygenase 1 (IDO1), and PD1 (Diesendruck and Benhar, 2017) expression were significantly upregulated (*p* < 0.0001) in the CDC42 high expression glioma patients (Figures 8A, B). Through correlation analysis, we discovered that the expression levels of CDC42 and PD-L1 in the TCGA (*R* = 0.48, *p* < 0.0001) and CGGA (*R* = 0.49, *p* < 0.0001) datasets exhibited a certain positive association (Figures 8C, D). Treg cell infiltration in glioma inhibits CD8^+^ T lymphocytes (CTLs) antitumor activity and may mediate resistance to ICIs (Amoozgar et al., 2021). We investigated the associations between CDC42 expression and several Treg cell markers, and we discovered that CDC42 expression was positively correlated with CD4 (*R* = 0.575, *p* < 0.0001), IL2RA (CD25, *R* = 0.551, *p* < 0.0001), and IL7R (CD127, *R* = 0.513, *p* < 0.0001) expression levels in the TCGA database (Figures 9A, C, E). The results were validated in the CGGA database (Figures 9B, D, F). All evidence suggests that CDC42 may be a target for glioma immunotherapy.

## 4. Discussion

Glioma is the most common and deadliest tumor of the nervous system (Touat et al., 2017), and even with the implementation of various treatments such as surgery, radiotherapy, and chemotherapy, the treatment of glioma has not achieved satisfactory results. The advancement of immunotherapy, such as immune checkpoint inhibitors (ICIs), chimeric antigen receptor T-cell (CAR-T) therapies, and adoptive cell therapies (Kennedy and Salama, 2020), guides the current research toward actively exploring new immunotherapy targets. ICIs have demonstrated some effectiveness in the immunotherapy of non-small cell lung cancer, breast cancer, and colorectal carcinoma (Adams et al., 2019; Morse et al., 2019; De Ruysscher et al., 2022). Anti-PD1 medicines, which are a kind of immunotherapy, have demonstrated potential in glioma preclinical research (Reardon et al., 2016; Kim et al., 2017); however, the majority of clinical trials have not achieved the anticipated levels of effectiveness (Caccese et al., 2019). Therefore, aggressively seeking novel targets is crucial for increasing glioma patient survival.

CDC42 is a signaling molecule with GTPase activity in the Rho family, which is associated with regulating the dynamic tissue and membrane transport of the cytoskeleton to promote physiological processes such as cell proliferation, motility, polarity, cell division, and cell growth (Ridley, 2001; Melendez et al., 2011). The upregulation of the expression of CDC42 is closely related to the development and metastasis of gastric cancer, breast cancer, and lung cancer (Du et al., 2016; Cruz-Collazo et al., 2021; Yao et al., 2021). Yang et al. (2022) observed that the silencing of CDC42 effector protein 3 (CDC42EP3) significantly limited the proliferation and migration of tumor cells.

Through online databases, we discovered that CDC42 was enriched in numerous tumor tissues. High expression of CDC42 in glioma patients was positively correlated with poor prognostic factors (higher WHO grade, IDH wild-type, MGMT non-methylated status, and 1p19q non-codeletion status). The KM curves demonstrated that the survival time of glioma patients in the CDC42 high expression group was shorter than the CDC42 low expression group. The multivariate Cox analysis pointed out that CDC42 was a poor independent prognostic factor for glioma patients. The ROC curves (AUC > 0.7) in the TCGA database predicted that the 1, 2, and 3 year survival glioma patients and some outcome discrepancies in the CGGA database may be attributed to the removal of certain patient records with insufficient survival information.

Functional enrichment analysis revealed that the enrichment of CDC42 was connected to several processes that encouraged the development of gliomas, including some immune and inflammatory responses. Tumor microenvironment (TME) referred to as the complex and abundant multicellular environment, including immune cells in tumor formation (Bejarano et al., 2021), and there was a difference in the proportion of immune cells in CDC42 high expression and low expression glioma samples using the ssGSEA and CIBERSORT algorithms. For instance, both algorithms showed a considerable enrichment of Treg cells in samples of glioma with high CDC42 expression. Treg cells in the tumor microenvironment can restrict the activation and development of CD4 helper T cells and CD8 cytotoxic T cells via a variety of mechanisms (Li and Rudensky, 2016; Newton et al., 2016), resulting in diminished tumor antigen-expressing responsiveness and immunological escape (Cuadrado et al., 2018; Campbell and Rudensky, 2020). These findings indicate that CDC42 hurts antitumor immunity in glioma.

As immune system controllers, immune checkpoints are essential for maintaining autoimmune tolerance and controlling the intensity and duration of immune responses in peripheral tissues. However, tumors can take over these pathways and activate them repeatedly, impairing antitumor immunity and encouraging carcinogenesis (Kalbasi and Ribas, 2020). Therefore, immune checkpoint inhibitors play a crucial role in tumor immunotherapy. As a well-known immune target, immunotherapy with the blockade of PD1 and its ligand PD-L1 can improve the survival time of patients with lung cancer (De Ruysscher et al., 2022) and colorectal cancer (Andre et al., 2020). Our study found that several immune checkpoints (BTLA, CD200R1, PD-L1, B7-H3, CD70, CTLA4, IDO1, and PD1) in patients with CDC42 high expression glioma were upregulated compared to the CDC42 low expression group. The expression of CDC42 was positively correlated with the expression of PD-L1. As mentioned earlier, Treg cells play an important role in the immune escape of tumors. CDC42 had a weaker correlation with several Treg cell markers (CD4, CD25, and CD127) in the CGGA database than in the TCGA database, we assumed that the variation in data presentation was attributable to the ethnicity of the CGGA samples database, which requires a large amount of data to further prove.

Although the relationship between CDC42 and a variety of cancers in multiple databases was integrated, and the expression characteristics of CDC42 in glioma were also analyzed, the study still has certain limitations. *In vivo* and *in vitro* experiments are needed to confirm the findings and to conduct additional mechanistic research. In conclusion, the prognoses of glioma patients are influenced by CDC42, which is associated with immune infiltration, and CDC42 could be an immunotherapy target for glioma. The treatment of gliomas and other types of tumors may benefit from these findings.

## Data availability statement

The original contributions presented in the study are included in the article/Supplementary material, further inquiries can be directed to the corresponding authors.

## Ethics statement

Ethical review and approval was not required for the study on human participants in accordance with the local legislation and institutional requirements. Written informed consent from the patients/participants or patients/participants' legal guardian/next of kin was not required to participate in this study in accordance with the national legislation and the institutional requirements.

## Author contributions

TJ: article design, conception, data acquisition, data collation, statistical analysis, software, picture drawing, investigation, and writing—review and editing. XW and JH: investigation and writing—review and editing. DC: final approval and writing—review and editing. All authors contributed to the article and approved the submitted version.

## Supplementary material

Supplementary Figure 1 Association between CDC42 expression and pathological classifications of glioma.

Supplementary Figure 2 Top 20 CDC42-binding proteins from the string database. **(A)** Top 20 CDC42-binding proteins network. **(B)** Top 5 proteins that pass MCC screening, and darker colors indicate higher rankings.

Supplementary Figure 3 Top 20 upregulated and downregulated genes' heatmaps in the TCGA database.

Supplementary Figure 4 Top 20 upregulated and downregulated genes' heatmaps in the CGGA database.

Supplementary Table 1 Twenty-nine immune-associated gene sets.
