# Supplementary figures and images for: CDC42—A promising immune-related target in glioma
Source: Front Neurosci. 2023 Jul 5;17:1192766. doi: 10.3389/fnins.2023.1192766 (PMC10354248; doi:10.3389/fnins.2023.1192766)

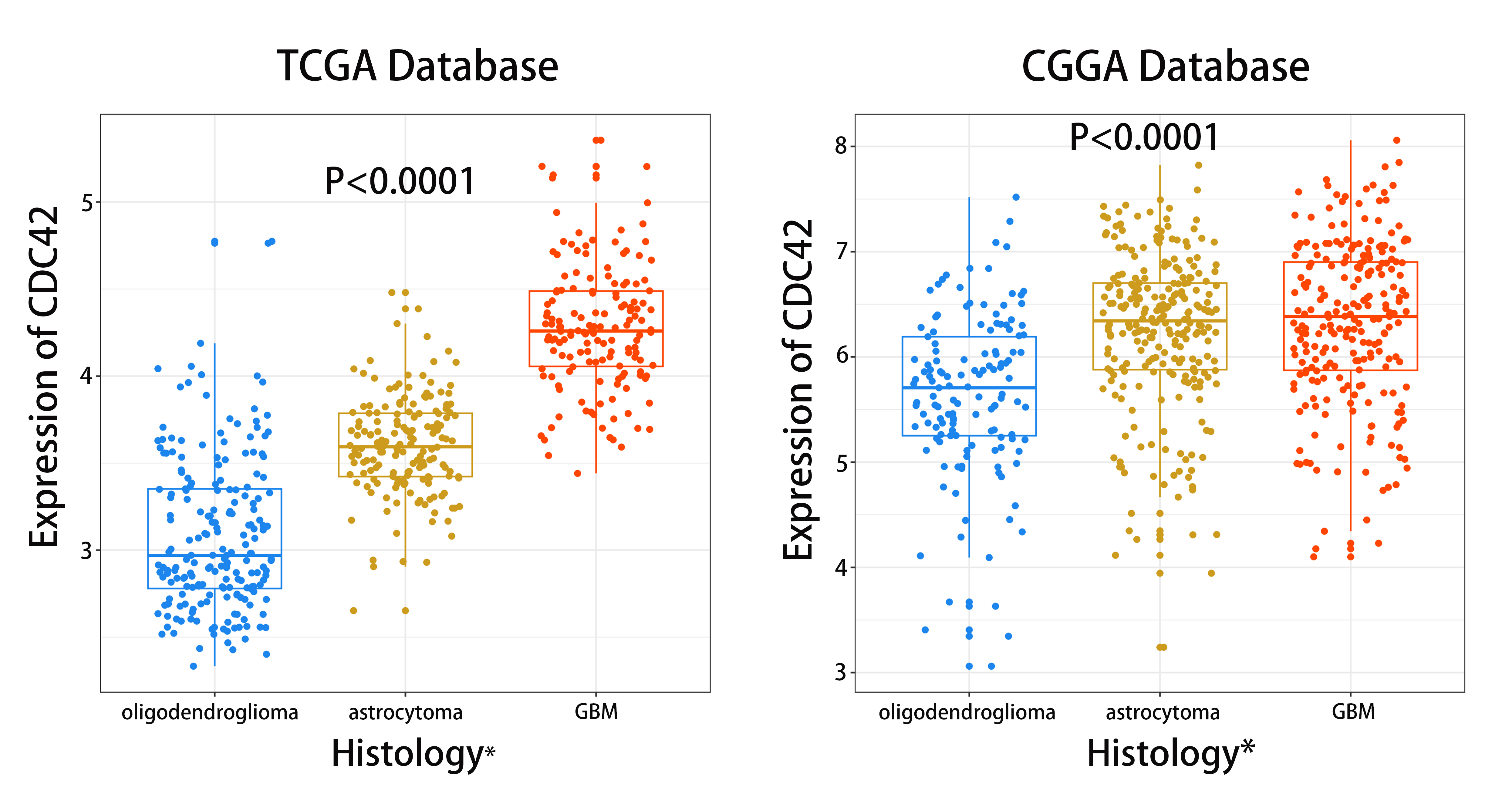

Supplement: Supplementary Figure 1 — Association between CDC42 expression and pathological classifications of glioma. [file Image_1.TIFF]

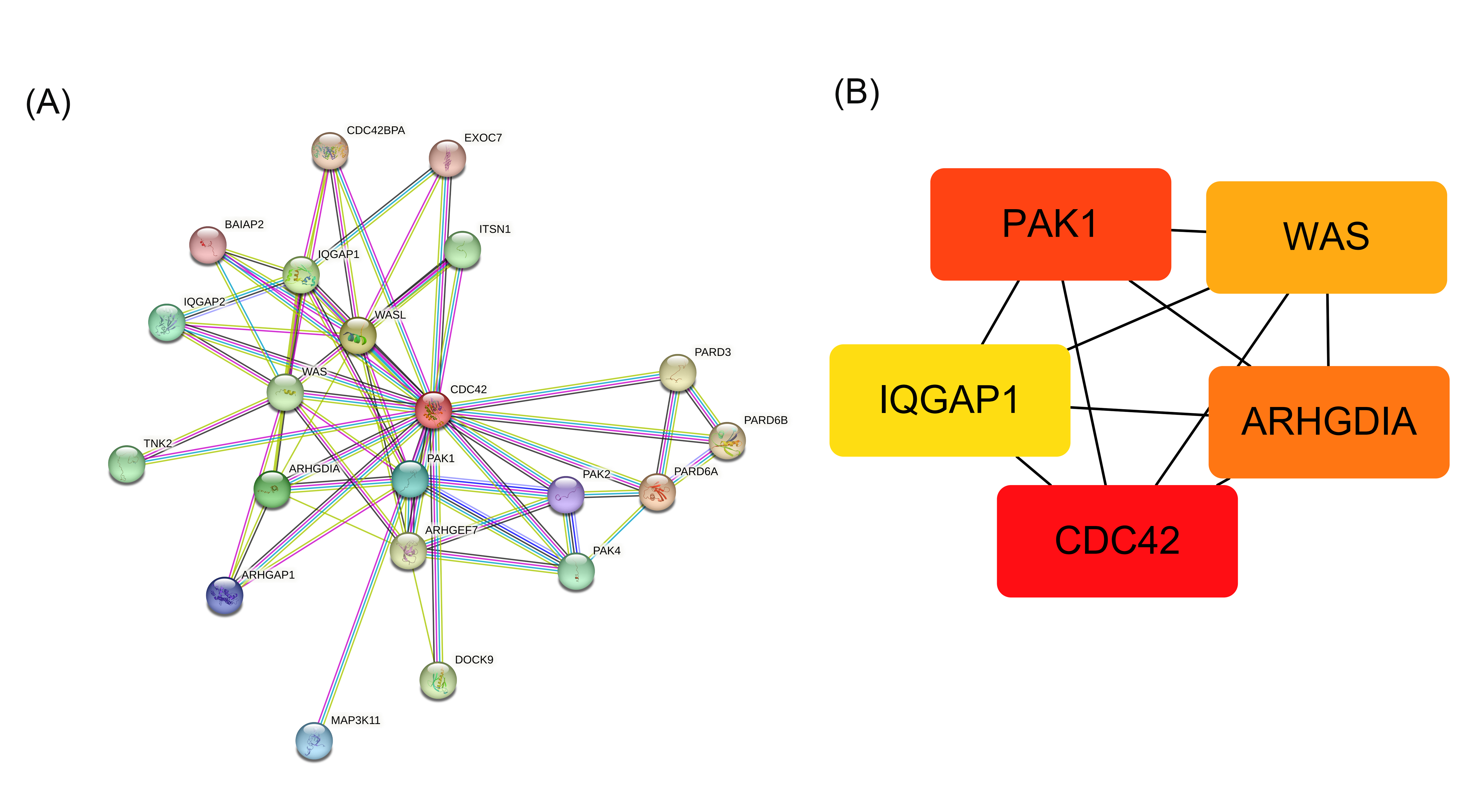

Supplement: Supplementary Figure 2 — Top 20 CDC42-binding proteins from the string database. (A) Top 20 CDC42-binding proteins network. (B) Top 5 proteins that pass MCC screening, and darker colors indicate higher rankings. [file Image_2.TIFF]

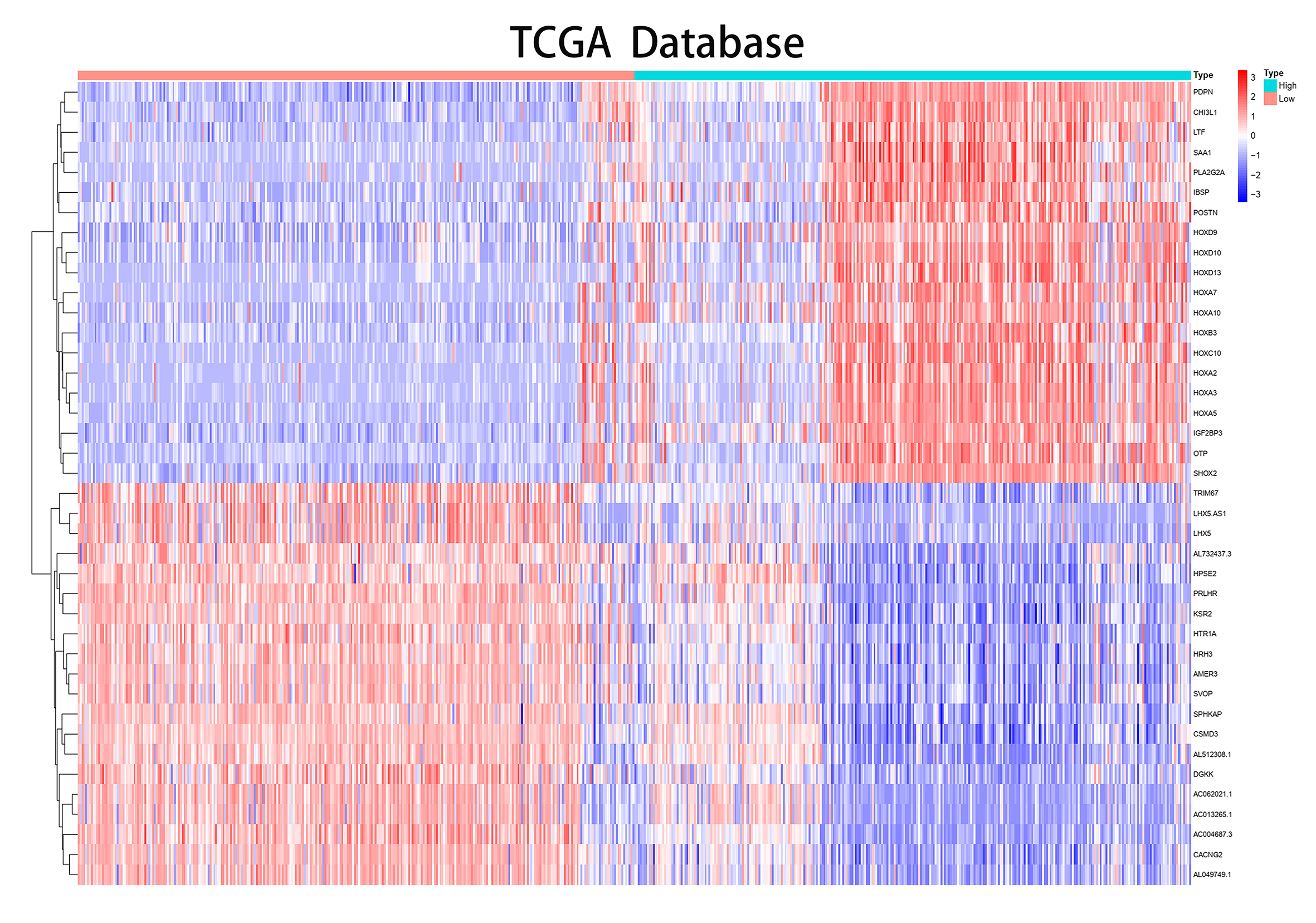

Supplement: Supplementary Figure 3 — Top 20 upregulated and downregulated genes' heatmaps in the TCGA database. [file Image_3.TIFF]

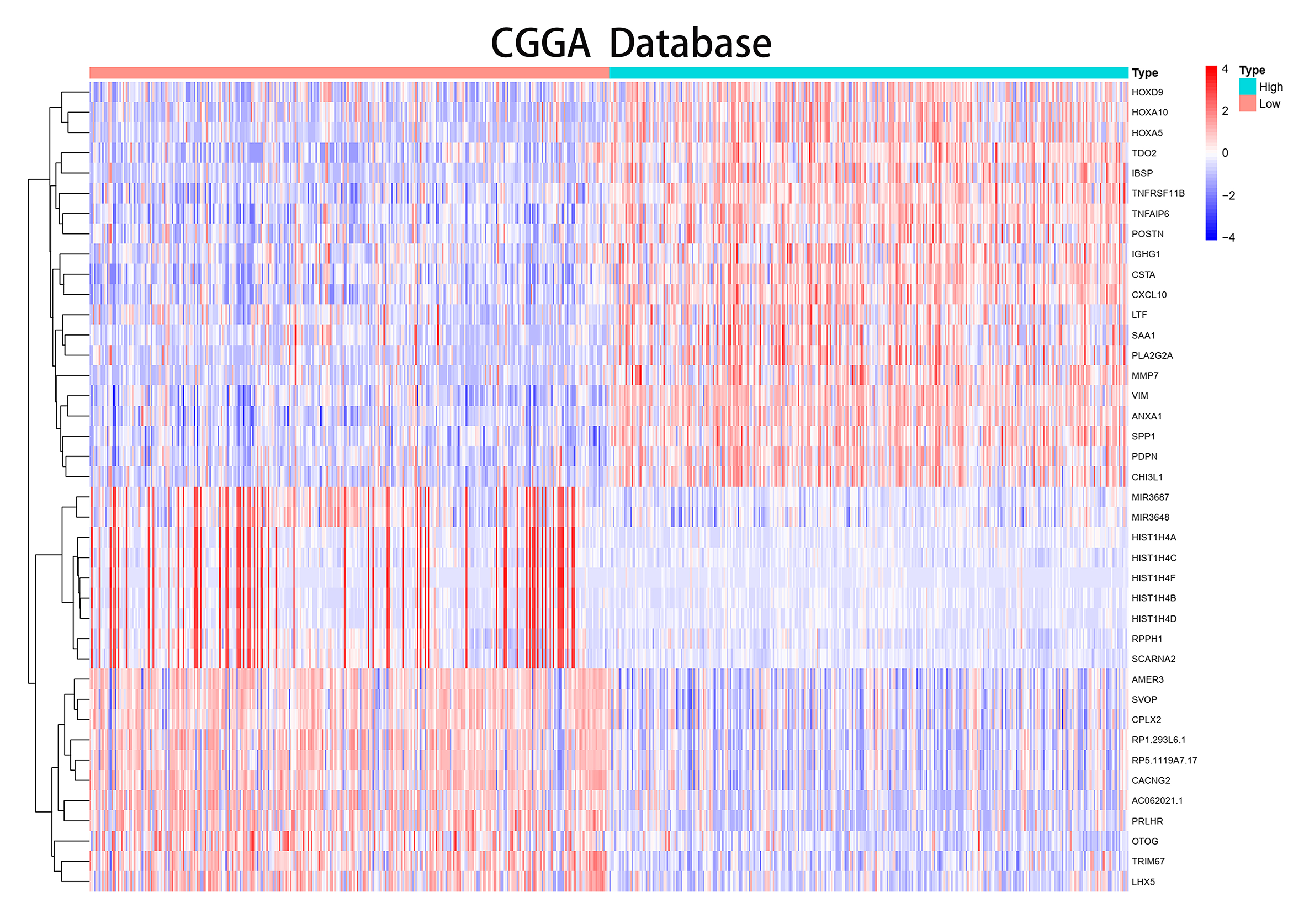

Supplement: Supplementary Figure 4 — Top 20 upregulated and downregulated genes' heatmaps in the CGGA database. [file Image_4.TIFF]
